# Exploring the Effectiveness of Cooperative Pre-Service Teacher and Generative AI Writing Feedback on Chinese Writing

**DOI:** 10.3390/bs15040518

**Published:** 2025-04-13

**Authors:** Hongli Yang, Yu Zhang, Jixuan Guo

**Affiliations:** 1 Faculty of Education, Northeast Normal University, Changchun 130024, China; zhangyu622@nenu.edu.cn; 2 International School of Business and Finance, Sun Yat-sen University, Zhuhai 519082, China; guojx68@mail2.sysu.edu.cn

**Keywords:** writing feedback, generative AI, pre-service teacher, cooperative writing feedback

## Abstract

Due to their efficiency, stability, and enhanced language comprehension and analysis capabilities, generative AIs have attracted increasing attention in the field of writing as higher-level automated writing evaluation (AWE) feedback tools. However, few studies have examined the impact of pre-service teachers using generative AI in combination with their own teaching experience to provide feedback on Chinese writing. To fill this gap, based on 1035 writing feedback texts, we examined the differences in writing feedback between 11 pre-service teachers and Erine Bot (a generative AI) and interviewed the pre-service teachers about their willingness to cooperate with generative AI. The collaborative writing feedback generated by the pre-service teachers using AI was compared with the feedback generated by the pre-service teachers and generative AI separately. We identified that, although Ernie Bot provided significantly better feedback than the pre-service teachers in three specific areas (except for language expression), and both Ernie Bot and the pre-service teachers had respective advantages in terms of writing strategy, human–computer cooperative writing feedback was significantly better than the writing feedback provided by either Ernie Bot or the pre-service teachers alone. The was true across all aspects of the feedback in terms of focus and strategy. These findings can support the training of pre-service teachers and improve the writing quality of their students via implementing AI to provide more effective writing feedback.

## 1. Introduction

With the rapid development of information technology, intelligent technology is gradually penetrating the field of education, providing infinite possibilities for innovative teaching methods. Among these methods, generative AI, with its powerful natural language processing ability, has changed the means of providing feedback in the teaching of writing and has become an increasingly efficient and user-friendly writing feedback tool (Wambsganss et al., 2022; M. Zhu et al., 2020). However, despite the many theoretical advantages of generative-AI-based feedback, there is a lack of in-depth research on the effectiveness of its practical application in the field of language writing for primary school students. Cultivating primary school students’ writing skills is a critical objective in foundational education, profoundly shaping the students’ development in verbal expression, logical thinking, and emotional articulation (Yao, 2013). In this process, teacher feedback plays a crucial role, not only helping students to identify and correct errors in their writing but also promoting the improvement of their writing skills and development of their thought processes (Bitchener & Storch, 2016; Cheng et al., 2021; Schuldt, 2019; Graham et al., 2015). However, in practice, teacher feedback is often affected by a variety of factors, such as personal subjective factors, time, and energy, and it is difficult to ensure accurate and consistent feedback (L. Huang, 2009). In contrast, generative AI can provide more objective and accurate feedback through big data analysis and algorithmic optimization (Crompton & Burke, 2023). At the same time, generative AI can also quickly process a large amount of student work, saving teachers a lot of time and improving their work efficiency (Mizumoto & Eguchi, 2023). However, despite the advantages of generative AI feedback, there are still many controversies regarding its use as a complete replacement for teacher feedback. Firstly, the feedback from generative AI cannot completely replace human interactions and the role of teachers in developing social and emotional skills. Human interactions play a crucial role in building relationships, empathy, and collaborative learning, which AI may struggle to replicate (Haleem et al., 2022). Secondly, how to effectively utilize the feedback from generative AI to improve the quality of teacher feedback while respecting and responding to students’ differentiated needs and providing them with more accurate writing guidance is also an urgent issue to be solved. Therefore, this study aims to explore the differences in the feedback given by generative artificial intelligence and teachers in regard to primary school students’ Chinese writing (the Chinese curriculum standards for compulsory education of the People’s Republic of China highlight that Chinese writing should be specific and clear according to different needs and that the writing text should express students’ experiences and ideas in a literal way) and also attempts to explore the possibility of using generative AI as a feedback aid to improve the quality of feedback and writing. Finally, it is important to note that we chose pre-service teachers for the following reasons. According to Foucault’s “regime of truth” (2000), as the universal politics of social truth, the discourse of rights in the social system (the discourse system constructed by rights relations, including rules and norms, etc.) may both constrain and motivate teachers (Ball, 2013). For pre-service teachers, on the one hand, the discourse of rights provides limitations, for example, pre-service teachers should use new technologies to assist their teaching; on the other hand, pre-service teachers are in the stage of training and formation, eager to become excellent teachers, and may be more willing to follow such rules, demonstrating the power of applying new technologies.

## 2. Literature Review

### 2.1. Writing Feedback and Analysis Framework

In the context of writing instruction, feedback refers to “the input from the reader to the writer, whose function is to provide the writer with information for revising the composition” (Keh, 1990, p. 294). Writing feedback plays a crucial role in writing instruction. It is not only a bridge of communication between teachers and students but also an important way for students to improve their writing skills and enhance their creativity (Pourdana & Asghari, 2021). Numerous studies have shown the importance of feedback in writing instruction (Radecki & Swales, 1988; Shi, 1998). Writing feedback helps to increase students’ motivation and attention towards writing. By obtaining comments and suggestions from others, students can feel their progress and achievement, thus stimulating interest in writing (Li & Wu, 2005). Professional feedback from teachers, instant feedback from intelligent systems, and peer-to-peer assessment can all contribute to the development of students’ writing skills to varying degrees. After receiving feedback, students can gradually improve their writing skills through continuous reflection and adjustment.

However, the key to giving feedback lies in determining which aspects to focus on and which strategies to employ. Accordingly, some scholars (e.g., Truscott, 1996; Ferris & Roberts, 2001; Chandler, 2003) have found that feedback focus and strategy are the key factors affecting the effectiveness of writing quality and writing outcomes. Feedback focus primarily refers to which writing issues the feedback is centered on, whereas feedback strategy involves the manner in which teachers provide feedback. Regarding feedback focus, most of the existing studies have focused on English writing feedback, including three aspects: content, organization, and language (e.g., Alshuraidah & Storch, 2019; Yang et al., 2006). Considering the context of Chinese writing instruction (Chinese writing is closely related to Mandarin, as it involves expressing thoughts and ideas in written form using the Mandarin language) and following Chinese curriculum standards, textbooks, and writing evaluation standards, four aspects were identified as suitable focuses for writing: theme development, writing framework, language expression, and text presentation. Therefore, this study adopted these four indices to reflect the focus of the feedback.

Regarding feedback strategies, Day et al. (1984) pointed out corrective feedback, which is a type of feedback that is specifically targeted at language errors. Non-corrective feedback refers to a form of feedback that does not directly point out mistakes or provide corrective suggestions in the feedback process. In language learning and teaching, non-corrective feedback mainly reflects positive evaluations of learner performance and recognition of a learner’s effort rather than directly pointing out errors (Nunan, 1991). Since non-corrective feedback does not directly point out errors, it can provide some constructive suggestions to help the learner develop further. This feedback, which focuses on the positive aspects of the learning process rather than solely on the outcome, can inspire intrinsic motivation in students and make them more willing to continue their efforts and exploration (Chai, 2024). In addition, Ellis et al. (2008) and Sheen et al. (2009) categorized corrective feedback into focused feedback and unfocused feedback based on the range of the feedback focus. Overall, corrective feedback and non-corrective feedback are the mainstream classifications of feedback strategies; therefore, corrective and non-corrective feedback were taken as the feedback strategy framework in this study.

### 2.2. Generative Artificial Intelligence and Writing Feedback

With the continuous progress and development of computer technology, the application of technology in the field of teaching and assessment has become increasingly sophisticated. One of the key applications is automated writing evaluation (AWE) feedback. Automated writing evaluation (AWE) feedback is a cost-effective and efficient alternative to manual assessment and feedback, significantly reducing the labor required for test assessment (Zhang et al., 2016). Automated writing evaluation (AWE) feedback can provide a wide range of feedback for student compositions, such as grammatical errors (Warden & Chen, 1995), vocabulary revision (Huang & Zhang, 2018), and content, organization, and linguistic suggestions (Burston, 2001). With the expansion of class sizes, as well as students’ expectations for personalized feedback and fair and objective assessments, the application of automatic assessment systems for composition has become increasingly widespread. The most popular automated writing assessment systems include “Criterion”, developed by the Educational Testing Service (ETS), “ProWritingAid”, founded in London, UK, and “Write & Improve” developed by Cambridge University. A common automated writing assessment system used in China is “www.pigai.org”, developed by the Beijing WordNet Technology Company. This system allows learners to continuously participate in the “feedback–practice–feedback” cycle of essay writing, thus improving their essay writing skills.

Automated writing evaluation (AWE) feedback, often referred to as computer-generated feedback, has been shown to offer significant advantages over manual revision, including immediate comments on student writing (Dikli, 2006), multiple revision opportunities (Warschauer & Ware, 2006), and the availability of overall and analytic scoring (Shermis & Burstein, 2003). AWE feedback can help learners to solve language-related problems in writing (Z. Li et al., 2014) and allow student writers to experience greater learner autonomy (Y.-J. Wang et al., 2013). Studies have shown that as students make diligent revisions based on automated feedback on their compositions, their writing performance improves (M. Zhu et al., 2020; Kellogg et al., 2010). Researchers have also found that AWE feedback had a positive impact on writing accuracy (J. Li et al., 2015; Z. Li et al., 2017). AWE systems have both advantages and disadvantages, and we should treat them critically. Due to its high efficiency in correcting students’ papers, an AWE system can reduce the workload of teachers, so that teachers can focus on the feedback content and feedback strategies (Z. Li et al., 2014; Zhang & Hyland, 2018). Meanwhile, we also need to critically examine the potential limitations of AWE systems. Specifically, we should use an AWE system critically because of its dual limitations, namely, the limitation of the system itself in that it cannot recognize multimodal, context-specific, and sociocultural contexts (Vojak et al., 2011) and the limitation in that it cannot guide students to a deep inspiration for content depth and logical coherence (Cheville, 2004) or personalized expression (Stevenson & Phakiti, 2014).

With the advancement of complex language processing technology in recent years, generative AIs such as ChatGPT, ERNIE Bot, Bard, Stable Diffusion, and Dall-E have attracted more and more attention in the field of writing. Generative AI not only inherits the efficiency and stability of automated machines but also has more powerful language understanding and analytical ability via the introduction of technologies such as deep learning and natural language processing. It is able to mimic human writing styles and thought patterns, analyze and evaluate texts in greater depth, and thus provide more comprehensive and accurate assessment and feedback on learning (Crompton & Burke, 2023). Generative AI can provide feedback on a student’s performance by grading their written assignments (Landauer, 2003). Mizumoto and Eguchi (2023) validated the reliability and accuracy of ChatGPT’s writing feedback by using it as an automated essay grading tool and showed that ChatGPT reduced the time required for grading, increased the efficiency of writing feedback, and was able to provide immediate grading and feedback on students’ writing skills. In addition, Dai et al. (2023) used ChatGPT to provide corrective feedback on undergraduate writing and found that Gen AI’s feedback was more readable and detailed than the instructor’s feedback. Su et al. (2023) stated that in an argumentative essay writing environment, ChatGPT can support argumentative essay writing from the perspectives of structure, language, and content and also provide adaptive feedback for students’ argumentative writing problems, including low-level language problems and high-level problems regarding the organization of arguments, with adaptive and differentiated assistance. These studies have demonstrated the feasibility and reliability of using Gen AI tools such as ChatGPT and ERNIE Bot for automated writing feedback.

However, generative AI has many known and unknown limitations that need to be considered before using it as an AWE tool. For example, generative AIs are unable to assess the validity of content and determine whether the output they generate contains false or erroneous information, so their use requires human supervision (Lubowitz, 2023). Moreover, over-reliance on generative AI tools is detrimental to students’ writing skills development (Warschauer et al., 2023), and students tend to accept information directly from the AWE tools without validating them (Koltovskaia, 2020). Most of the previous research on writing feedback has been conducted from a single perspective of teacher feedback or AI feedback, and few studies have explored the differences between the two from a comparative perspective. Especially in the Chinese context, although there have been some studies on the use of generative AI such as ChatGPT in writing feedback, they have mainly focused on its chat function, and in-depth exploration of writing feedback is still insufficient (Murugesan & Cherukuri, 2023). To fill this research gap, we will use parametric tests to explore the differences between generative AI feedback and teacher feedback in a primary school language writing course in depth and try to explore the possibility of using generative AI feedback as a teaching aid to promote the quality of teacher feedback. Considering the appropriateness of language diagnosis and expression, we will take ERNIE Bot 3.0 as the generative AI tool in this study.

### 2.3. Pre-Service Teachers and Writing Feedback

Teacher professional development is an important topic in the field of education. It is an ongoing process that encompasses pre-service and in-service training throughout a teaching career (Xiao, 2002; L. Zhang, 2018). The professional growth of pre-service teachers, as the fresh talent of the teaching force, has received extensive attention (Van Katwijk et al., 2023; Huan et al., 2020). Several studies have shown that the training experience of pre-service teachers studying at Teacher College has a significant impact on their future teaching careers. In addition to acquiring solid professional knowledge, pre-service teachers also need to develop teaching skills and the ability to communicate effectively with students (Xie & Xiong, 2014; Copland, 2010). Related studies on the professional development of pre-service teachers have focused on how pre-service teachers develop an identity as educators through education and practice and the impact of this process on their teaching practice and professional development (Y. Huang, 2021; C. Zhang, 2016). Other studies have also emphasized that analytical reflection serves as a key mechanism for teachers’ professional development, helping pre-service teachers understand their teaching practices and make improvements to them (Han & Wang, 2008; Leng et al., 2020).

There are obvious differences between pre-service teachers and expert teachers in their professional development. As they are at the start of their teaching careers, pre-service teachers are in a critical period of accumulating teaching experience and improving their teaching skills. They have a unique dual role: On the one hand, they are college students receiving higher education. Under the guidance of their tutors, they systematically study curriculum standards, gain a deep understanding of the teaching objectives and content requirements of various disciplines, and master professional knowledge such as teaching theory, teaching design, teaching methods, and teaching evaluation. On the other hand, they are also student teachers who are about to enter educational practice. Their training program covers a wide range of educational practices, including receiving detailed guidance from practical tutors in primary schools, actively participating in teaching practice and class management, and working closely with practical tutors to jointly promote the growth of their professional ability. Under this training model, even though pre-service teachers have not accumulated rich teaching experience in the internship stage, their attention to the learning process of students remains fresh and highly focused, which enables them to observe the growth trajectory of students and the challenges they face in detail. They tend to give students more detailed and patient feedback (Kong & Wu, 2013). However, it is worth noting that these behavioral characteristics of pre-service teachers are not entirely determined by their personal attitudes or personality traits, but more influenced by the environment in which they work and the education and training they have received. Foucault (2000) proposed the concept of a “regime of truth” to refer to a kind of “universal politics of truth” in society or a system of discourse in which people participate in various cultural contexts. As a subsystem of society, the education system also has authoritative and influential rules, norms, and concept systems. This kind of power relationship defines the behavior mode, value orientation, and principles, which in turn influences the behavioral characteristics of the teacher (Karnovsky, 2020; Karnovsky et al., 2022). In particular, due to the development of technology, requiring teachers to use technology to assist teaching has become the discourse rule of the education system. For pre-service teachers trained in Teacher College, they must abide by the discourse power of the education system by using specific curricula and activities, thus embracing the idea of technology as a teaching aid and putting it into practice. However, from another point of view, the discourse of rights provides a development opportunity for pre-service teachers. In order to become better teachers, pre-service teachers are more likely to be willing to follow the regulations stipulated by this discourse of rights and may respond positively to the requirements of technological empowerment, actively learn new educational technologies, and integrate them into teaching practices. As a result, they may be more likely to accept new technology and methods, making writing feedback more innovative and flexible. In the highly integrative professional field of teaching, the accumulation of specialized knowledge often stems from an individual’s in-depth practice in a particular teaching environment. Lehmann et al. (2019) explored the effects of writing tasks and prompts on the integration of specialized knowledge among pre-service teachers through an experiment. The results of the experiment showed that, through writing feedback, pre-service teachers were able to integrate and apply their learned professional knowledge more effectively, thereby enhancing the quality of their teaching. As emphasized by some researchers (Berliner, 2004; Chi, 2011; Gold et al., 2016; Prilop et al., 2021), experienced teachers demonstrated richer expertise in their teaching practices. However, compared to in-service teachers, pre-service teachers have not yet really entered into educational practice and appear to have relatively limited opportunities for systematic learning and practicing teaching. Holstein et al. (2022) explicitly pointed out that pre-service teachers may have difficulties in accurately assessing students’ writing levels and needs due to their lack of rich teaching experience, thus affecting the effectiveness of feedback. In the face of this challenge, the rise of generative AI offers new possibilities for optimizing pre-service teachers’ feedback mechanisms. In particular, large-scale language training models, represented by Ernie Bot and ChatGPT, have shown unprecedented advantages in text feedback. These models are not only able to quickly analyze students’ work, but also provide precise and targeted feedback, which provides a valuable reference for pre-service teachers. Because of such advantages, we can use AI as a tool to generate writing feedback; therefore, in this study, we used Ernie Bot as an experimental tool on the basis of asking teachers’ wishes.

Currently, the field of research on teacher feedback is undergoing an increasing change. The binary framework of traditional research, which divides teacher feedback and automated feedback, has shown its limitations and cannot meet the needs of modern education. Human–machine collaboration is gradually becoming a new research trend (Han & Li, 2024; F. Wang et al., 2024). In this context, this study compares and analyzes the feedback of pre-service teachers with that of generative AI, aiming to explore a new man–machine collaboration model that can make full use of the efficiency and precision of AI while retaining the educational wisdom and humanistic care of pre-service teachers. We look forward to seeing more research and practice of human–machine collaborative teaching and learning with generative AI as a powerful tool in the future, so as to jointly promote the progress and development of education.

## 3. Human–Machine Collaboration as the Theoretical Foundation

Human–machine collaboration theory originated from the exploration of a highly intelligent man–machine interaction system. X. Qian et al. (1990) first proposed the concept of “comprehensive integrated engineering”, which laid the basic framework of human–machine collaboration. Later, Lenat and Feigenbaum (1991) further proposed that computers and humans can be colleagues, each doing what they are good at, which greatly promoted the development of human–machine collaboration theory. The “manplus-machine” concept, proposed by Kasparov after his 1997 game against “Deep Blue”, subverted the traditional human–machine confrontation thinking: the player can adopt the AI advice but retain the final decision-making right, just as the driver maintains the autonomy of route selection with the assistance of GPS navigation (Kasparov, 1997). It can be seen that no matter how man and machine cooperate, it is always man who plays the decisive and leading role; however, the auxiliary role of the machine has been changing somewhat. The early computer, as an executive tool without autonomous consciousness, was completely subject to the preset programming of human beings. Contemporary AI has autonomous learning and decision-making capabilities, can provide personalized support according to user needs, and has become a learning partner and work partner of humans (Wang, 2019). The core idea of human–machine collaboration theory is to realize the intelligence of human–machine integration, rather than simply adding human intelligence and machine intelligence. In the process of human–machine collaboration, as an auxiliary tool, machines can take on tedious, repetitive tasks, while in the leading role, humans focus on tasks that require creativity, judgment, and emotional investment, thus achieving the best collaboration between humans and machines. Human–machine collaboration theory provides a new perspective and thought for the development of education in the intelligent age. Zheng et al. (2024) point out that AI models can provide immediate and targeted corrective feedback, which is particularly important for the revision of writing. Rad et al. (2023) proposed that AI feedback could be used as an initial screening tool, allowing students to independently correct basic errors before submitting work for teacher review. This strategy effectively eases the teachers’ burden, allowing them to focus on advanced aspects of writing such as structure, argument, and style. At the same time, by using AI to provide feedback, teachers can provide students with personalized writing support to adapt to the needs of different students (Seo et al., 2021). In this study, human–machine collaboration theory provides a theoretical basis for exploring the collaboration model between AI and teachers.

## 4. Research Question and Method

### 4.1. Research Question

In this study, we tried to answer whether writing feedback from the cooperation of a pre-service teacher and generative AI will outperform the feedback from only a pre-service teacher or generative AI. Accordingly, the specific questions were as follows:

Q1: Are there differences between generative AI and pre-service teachers in terms of feedback focus (theme idea, writing framework, language expression, text presentation) and feedback strategies (corrective feedback and non-corrective feedback)? If so, what are the differences?
Q2: Are pre-service teachers willing to use generative AI to provide writing feedback?
Q3: Does the collaborative feedback of pre-service teachers using generative AI have advantages in terms of feedback focus and feedback strategies compared with that of only generative AI or only teacher feedback?

### 4.2. Method

#### 4.2.1. Participants

In this study, through the principle of voluntary participation, 11 pre-service teachers were recruited. All of them were postgraduate students in X university in China, majoring in Primary School Education, and from different provinces of China, such as Jilin province, Shandong province, He’ nan province, and Heilongjiang province. It should be noted that as students from X University, the 11 pre-service teachers will become primary school teachers after graduating. Meanwhile, during their studies at X University, they will experience two internships at a primary school, the first for one month and the second for two and a half months, during which they will work as student teachers and under the guidance of practicum supervisors who help student teachers practice teaching and manage the class. The 11 pre-service teachers recruited have all gone through two internships. As postgraduate students, all participants had experience of using generative AI and were more willing to use computers or new social media to learn and live during their studies.

#### 4.2.2. Procedure and Data Collection

**Figure 1:**
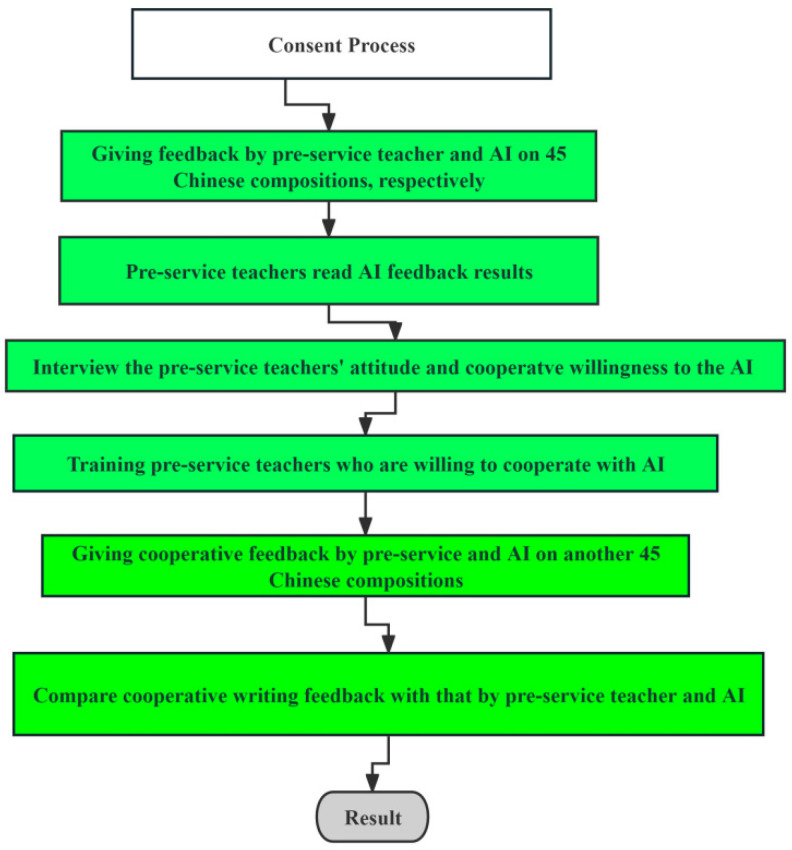
The research procedure.

In order to compare the advantages of the collaborative feedback provided by pre-service teachers using AI with that of only pre-service teachers or only AI, we first asked pre-service teachers and ERNIE Bot to give independent feedback on 45 Chinese essays, respectively. Then, pre-service teachers read the feedback results of AI and interviews were conducted to explore their willingness to cooperate with AI. Subsequently, we selected pre-service teachers who are willing to cooperate with AI to train until they were fully capable of using AI and were proficient in using AI for writing feedback; using AI, the pre-service teachers provided feedback on another 45 essays. Finally, the collaborative feedback was compared with that of only pre-service teachers or only AI to produce the results (see Figure 1).

Both quantitative methods and qualitative methods have been used in this study. Following the research procedure, firstly, quantitative data were obtained regarding writing feedback from pre-service teachers and AI, respectively. Considering the representativeness of the writing topic, we chose the one-unit proposition composition “My Favorite Thing” in Grade 5, which was edited by the Ministry of Education, PRC, asking students to choose an object they’d like to describe, while expressing their love for the object with their own emotions. According to the “Chinese Curriculum Standards for Compulsory Education” issued by the Ministry of Education of the People’s Republic of China in 2022, students in the fifth grade of primary school can express their true feelings in language. With a random sample of 166 compositions by fifth-grade students at an ordinary school in Sichuan Province, China, after screening, a total of 96 compositions of the same level (according to theme idea, writing framework, language expression, and text presentation) were obtained, 45 of which were used for the first writing feedback from the pre-service teachers and AI, respectively; the other 45 compositions were used for the second writing feedback (cooperative writing feedback from the pre-service teacher and AI). Therefore, 1035 writing feedback texts (45 texts written by AI + 495 texts (11 × 45) written by pre-service teachers + 495 texts (11 × 45) written cooperatively by pre-service teachers and AI) were obtained in this study.

In this study, writing feedback was given from the dimensions of writing focus and writing strategy based on the literature review. Firstly, four questions were asked to explore the writing focus, then writing strategy from corrective writing feedback and non-corrective feedback would be analyzed regarding the results of writing focus in this study. When collecting writing feedback from generative AI and pre-service teachers, we created four questions based on the focus of the writing. It is important to note that both generative AI and pre-service teachers were asked in the following order and only the first responses of the generative AI were collected: (1) From the perspective of theme idea, show your opinions and suggestions for the composition. (2) From the perspective of writing framework, propose your opinions and suggestions for the composition. (3) From the perspective of language expression, give your opinions and suggestions for the composition. (4) From the perspective of text presentation, put forward your opinions and suggestions for the composition. In order to avoid errors caused by the way the questions were asked, we used the same questions for every pre-service teacher and generative AI and there were no other prompts. Then, based on the results of writing focus from Ernie Bot and the pre-service teachers, we analyzed the writing strategies from the dimensions of corrective feedback and non-corrective feedback. In our study, to ensure trustworthiness for this study, peer examination was conducted (Merriam & Tisdell, 2015): two researchers analyzed the text of writing feedback using Linket 5 scores. At the beginning, the two researchers discussed the specific scoring criteria. After assigning a score to five writing feedback texts from generative AI and five writing feedback texts from pre-service teachers, we compared the scores and discussed the assessment criteria; our consistency was 0.921. Then, we discussed the different scores until agreement was reached.

In order to compare the differences in feedback from the pre-service teachers and AI, this study used SPSS 26.0 to perform the variation test. First, using SPSS 26.0, we conducted the normality test, then we performed independent-sample T tests on the writing focus and writing strategy feedback from Ernie Bot and the pre-service teachers, respectively, and judged the result according to Levene’s equal homogeneity test.

Then, qualitative data were obtained through interviews with pre-service teachers. The aim of the analysis was to investigate the pre-service teachers’ willingness to use Gen AI for writing feedback. After giving feedback and seeing the results of the generative AI, 11 pre-service teachers were interviewed using a structured interview with a total of four questions: What is your overall feeling about the writing feedback on generative AI? Specifically, how does AI respond in each dimension (theme idea, writing framework, language expression, text presentation)? Are you willing to use AI for writing feedback? And how to use AI for collaborative writing feedback? Structured interviews were conducted via WeChat phone and recorded with the consent of the interviewees. The interviewers asked each question one by one, and the 11 pre-service teachers answered them in order. Each pre-service teacher was interviewed for about 20 min, and the 11 teachers were interviewed for a total of 230 min. After the interview, we converted the interview recordings and tried to retain the interviewee’s original words. Regarding interview analysis, three-level coding was adopted because it not only comprehensively captured the depth of information but also constructed a solid theory from the bottom up, which significantly improved the rigor and explanatory power of the research. Through opening coding, axial coding, and selective coding, the results were generated. Each pre-service teacher’s interview text was coded with an index number for the year, month, and name (e.g., 2024:XJ—year 2024, XY’s name code). To ensure the inter-rater reliability (Gass & Mackey, 2000) of the processes of coding, two researchers conducted open coding, axial coding, and selective coding, respectively, and then discussed disagreements until reaching a consensus. Finally, 4 selective codes from 13 axial codes from 39 open codes were contracted by two researchers addressing pre-service teachers’ opinions and perceptions towards the AI feedback and their willingness to cooperate with AI.

To further test the pre-service teachers’ evaluation of writing feedback from generative AI, we asked the 11 pre-service teachers to assign scores to the feedback focus and feedback strategies for the writing feedback from generative AI on a scale of 1 to 5 (the higher the score, the better the rating). Overall, the pre-service teachers gave higher evaluations to the writing feedback from generative AI and were willing to try to perform writing feedback using generative AI. Based on this, we first trained 11 teachers to give feedback using generative AI until each pre-service teacher could use AI flexibly. Regarding the specific methods of cooperation between pre-service teachers and generative AI, all participants said that it was better to use generative AI for the initial feedback and then carry out the teacher feedback.

Finally, cooperative writing feedback on the remaining 45 compositions was performed by the pre-service teachers and AI together, and 495 cooperative feedback texts were obtained. To compare the differences between the cooperative writing feedback and the feedback from either only the pre-service teachers or only the AI, using SPSS 26.0 and MANOVA in general linear models, the three types of writing feedback were examined from the perspective of writing focus and writing strategy, respectively. We performed the Tamhane post hoc test because Levene’s variance homogeneity test was not equal. The effect sizes mainly included the sample number, means, SD, 95%CI, F value, and *p* value (according to the general *p* value, if *p* < 0.05, the effect is significant). Meanwhile, the writing feedback time with and without the cooperation of pre-service teachers and AI was tested via paired-sample *t* test.

## 5. Result

### 5.1. Differences in Writing Focus and Writing Strategy Feedback from Pre-Service Teachers and Generative AI

To examine the differences in writing focus feedback from pre-service teachers and generative AI, such as theme idea, writing framework, language expression, and text presentation, independent-sample T tests were conducted. Since the Levene test showed that each homogeneity of variance was unequal (*p* < 0.001), a two-tailed test that did not assume equal variance was taken as a *p*-value. As shown in Table 1, according to the mean value of writing focus, Ernie Bot outperformed the pre-service teachers in all aspects of writing focus. By further analyzing the data, we found that, except for language expression, Ernie Bot was significantly better than pre-service teachers in another three specific feedback focuses, identifying that generative AI might have advantages in writing feedback on Chinese composition compared to pre-service teachers (see Table 1).

Additionally, independent-sample T tests regarding the difference of writing strategy feedback from the pre-service teachers and Ernie Bot uncovered that both pre-service teachers and generative AI had their advantages, namely, generative AI (M = 3.333) outperformed pre-service teachers (M = 2.461) on the corrective feedback, with a significance at the 0.01 level, while the pre-service teachers were better than generative AI on non-corrective feedback, with a significance at the 0.001 level (see Table 2).

### 5.2. Pre-Service Teachers’ Opinions on AI Writing Feedback and Willingness to Cooperate with Generative AI

In order to examine the pre-service teacher’ opinions on the generative AI, 45 writing feedback texts constructed by generative AI were shown to the pre-service teachers. Since the feedback criteria for the pre-service teachers and the generative AI were exactly the same, after reading the generative AI feedback, the pre-service teachers were interviewed. Overall, the pre-service teachers were amazed at the speed and quality of writing feedback from generative AI and critically thought about AI writing feedback (see Table 3).

Despite some limitations in the feedback, such as a lack of flexibility, emotion, and individuation, pre-service teachers appreciated the effectiveness of writing feedback by generative AI. ”I was actually quite shocked, because I didn’t realize that generative AI was actually same as the teacher’s brain. In fact, for language teacher, the most difficult part of the teaching burden was to give feedback on children’s compositions. When conducting feedback, it especially consumed the teacher’s energy, and it was just something like a headache. But with the help of generative AI, feedback will become easier, because generative AI will reduce the workload of teachers by 80%” (2025-LS). Other language teachers agreed with LS and expressed similar opinions, “this is a good tool to reduce the work burden for all Chinese teachers because Chinese teachers teach Chinese subject as well as working as class teacher. The work of the class teacher has to spend a lot of our energy, meanwhile, Chinese teaching is not only writing, but also reading and others. Therefore, the job of a Chinese teacher was the hardest” (2025-YJ). Generally speaking, there are more than 40 children in a class in China, and each child’s composition has to be given feedback, so writing feedback has become a heavy burden for Chinese teachers. At the basic education stage in China, Chinese teachers also work as class teachers responsible for class management, class activities, the construction of class culture, and so on. The dual workload of teaching and class management places a heavy burden on Chinese teachers. “After reading the results of Ernie Bot, I am amazed, not only its speed, but its higher quality of feedback, which will help us reduce the work burden and improve the work efficiency” (2025-YJ). Through YJ, we knew that from the perspective of a pre-service teacher, generative AI would be helpful due to its importance in alleviating Chinese teachers’ work burden. Meanwhile, generative AI was able to give more specific suggestions for revising, more appropriate suggestions on the theme and emotion, more optimized sentences, correct sentences for problems, and so on. Therefore, all participant pre-service teachers were willing to cooperative with generative AI because cooperative writing feedback not only improved the efficiency and reduced the burden of writing feedback on Chinese teachers but also made the writing feedback warmer and more suitable for each student. In terms of how to cooperate with generative AI, pre-service teachers point out that teachers were more important than generative AI, where teachers were the subject and AI was the tool, “when teachers used AI for writing feedback, they should first establish an awareness that teachers should be the main and AI should be supplemented, and the auxiliary role of AI should be maximized” (2025-MY).

The use of AI could be used both in normal times and in special periods, such as when teachers do not know how to give feedback, when they are tired, and when they lack writing advice, and Chinese teachers could flexibly use the generative AI before, during, and after the feedback.

All of the pre-service teachers interviewed talked about the efficiency of generative AI. For the same 45 Chinese compositions, many pre-service teachers took an average of more than 12 min for a composition; however, the generative AI completed the feedback in less than one minute, which significantly saves time for writing feedback. Furthermore, generative AI could support more detailed and comprehensive feedback. For example, the pre-service teachers could only point out that the expression of the sentence was inappropriate, while the generative AI could give a specific and appropriate expression sentence. Regarding opinions on the writing feedback from generative AI, pre-service teachers rated it as follows (see Table 4).

Because the score ranged from 1 to 5, 2.5 was the average score. All scores were higher than 2.5, even reaching more than 4.6, identifying that pre-service teachers were willing to give writing feedback using generative AI. Through the interviews, we also learned that cooperative feedback could make up for the shortcomings of generative AI’s inability to provide emotional support, recognize some composition information, and understand students’ background information to provide personalized support, so cooperative feedback would be full of emotion, more comprehensive, and more personalized.

### 5.3. Differences Between Cooperative, Pre-Service Teacher, and Generative AI Feedback

To examine the differences in writing focus from cooperative feedback and pre-service teacher and Gen AI feedback, a difference test was conducted using MANOVA in General Linear Models. After the Tamhane post hoc test (see Table 5), based on the mean of the writing focus, we found that cooperative feedback significantly outperformed Ernie Bot and Ernie Bot significantly outperformed pre-service teachers in all aspects of writing focus.

To examine the differences in writing strategy from cooperative feedback and pre-service teacher and Gen AI feedback, a difference test was conducted using MANOVA in General Linear Models. After the Tamhane post hoc test (see Table 6), based on the mean of writing strategy, we found that though Ernie Bot might perform better than pre-service teachers in corrective strategy and pre-service teachers might have better effectiveness than Ernie Bot in non-corrective strategies, on the whole, cooperative feedback outperformed Ernie Bot and pre-service teachers in all aspects of writing strategy, with a 0.001 level of significance.

Regarding the feedback time, there was a significant difference with and without using cooperative writing feedback. The mean of the overall seconds spent on the 45 Chinese compositions by the 11 pre-service teachers was 494.273 without cooperation and only 27.273 s with Gen AI cooperation (see Table 7).

To further examine this point, we identified that there was a significant difference at a 0.001 level (see Table 8), suggesting that human–robot collaborative writing feedback significantly reduces the time taken to produce feedback.

## 6. Discussion

### 6.1. Overall Differences in Writing Focus and Writing Strategy Feeback from Pre-Service Teachers and Generative AI

Overall, ERNIE Bot significantly outperformed the pre-service teachers in all aspects of writing focus except for language expression and achieved this in a shorter period of time; this is similar to the results obtained for other automated writing assessment (AWE) tools (Wilson & Czik, 2016, p. 95). In the process of improving students’ writing ability and learning outcomes, the constructive feedback provided by teachers plays an important role. However, designing and implementing effective feedback strategies is a complex and challenging task that requires professional skills on the part of the teachers (Boud & Dawson, 2021; Graham, 2018). Despite the potential value of teacher feedback in improving student writing, in practice, the provision of feedback is often time-consuming, ineffective (Duijnhouwer et al., 2012; Kellogg & Whiteford, 2009), and uncertain (Parr & Timperley, 2010; Ryan et al., 2021), which may be related to the complexity of the feedback skills themselves and the diversity of students’ and teachers’ personal characteristics (Panadero & Lipnevich, 2022). Because pre-service teachers have not really stepped into the field of educational practice, they lack experience of writing feedback and have limitations from the gap between their understanding and the application of educational theory (Risan, 2020; la Velle, 2019). Additionally, pre-service teachers may be faced with multiple tasks, such as studying multiple courses, completing homework, and preparing for exams, at the same time during their learning stage at university (Yue et al., 2017). Consequently, it may be difficult for pre-service teachers to conduct detailed analysis and feedback on each student’s written work within a limited time, leading to a decline in the quality of feedback. Finally, from the perspective of cognitive psychology, pre-service teachers may be limited by their own cognitive load when dealing with student writing (Paas & van Merriënboer, 2020). They need to read, understand, analyze and evaluate students’ work in a short period of time, and this state of high cognitive load may affect their judgment and the accuracy of feedback.

Regarding feedback strategy, in terms of error correction feedback, ERNIE Bot was significantly better than pre-service teachers. Generative AI systems are often able to process and analyze large amounts of data, which allows them to analyze students’ essays in a short amount of time and find common grammatical errors, misnomers, and other problems. Through deep learning and natural language processing techniques, generative AI can quickly identify errors in learning material and cover even more small and common errors, especially those that even teachers might overlook. For primary school students, the correction of these details is very important to form good writing habits and improve language expression ability. In contrast, pre-service teachers may not be able to cover all possible types of errors due to their limited experience and knowledge. However, in terms of non-error correction feedback, pre-service teachers were significantly better than ERNIE Bot. The reason for pre-service teachers’ advantage in non-corrective feedback may be that the teaching methods experienced by pre-service teachers, who are often encouraged to stimulate interest during learning, result in more understanding of students’ emotional needs and emotional support (Rauduvaitė et al., 2015; Rahman et al., 2025) and holistic considerations such as students’ overall development, learning motivation, and classroom climate when they provide non-corrective feedback (Lipnevich et al., 2021). This is because pre-service teachers believe that corrective feedback may dampen students’ enthusiasm and initiative (Baron, 1988; Fang et al., 2021). The essence of pre-service teachers’ advantage in non- corrective feedback is the irreplaceability of the “human” in the educational process. This kind of feedback is not only a writing guide but also an emotional interaction. In the context of the growing popularity of AI tools, the important role of pre-service teachers in non-corrective feedback reminds us that the most moving education always takes place in the resonance of the mind beyond the reach of algorithms.

### 6.2. Pre-Service Teachers’ Willingness to Cooperate with Generative AI for Writing Feedback

Based on a structured interview, 11 pre-service teachers expressed their overall and specific perceptions about the writing feedback provided by generative AI. They expressed a willingness to cooperate with generative AI, even offering insights on how to effectively cooperate with AI to provide writing feedback. First, the 11 pre-service teachers were asked to think critically about the role of generative AI in providing writing feedback, both in terms of its advantages and disadvantages. The pre-service teachers were more likely to recognize the advantages, as opposed to the disadvantages, of generative AI for providing writing feedback, including saving time, reducing their workload, and providing specific suggestions. It must be acknowledged that generative AI has become an unstoppable force that is now widely used (M. Zhu et al., 2020). The perceptions of pre-service teachers were in line with the development trend of AI (Wambsganss et al., 2022). Additionally, all 11 pre-service teachers indicated their willingness to cooperate with generative AI in providing writing feedback, recognizing that it could complement their strengths and make the provided writing feedback more effective, comprehensive, personalized, and emotional. This further demonstrates the effectiveness and universality of the theory of human–machine collaboration (Wang, 2019). Finally, the pre-service teachers discussed the dynamics of cooperating with generative AI, stating that teachers should remain the authority and generative AI should be used as a tool to provide writing feedback. This helps to clarify the cooperative relationship when using generative AI to provide writing feedback. Based on the theory of human–machine collaboration and the interviews of 11 pre-service teachers, this study suggests adopting a teacher-led approach, with AI used as an auxiliary tool to provide writing feedback.

### 6.3. Differences Between Human–Computer Cooperative Writing Feedback and Ernie Bot Writing Feedback Alone and Pre-Service Teacher Writing Feedback Alone

By comparing the writing feedback from human–computer cooperation with the feedback from Ernie Bot or pre-service teachers alone, we found that the human–computer cooperative writing feedback was significantly better than that of Ernie Bot and the pre-service teachers in all aspects of feedback focus and feedback strategy. This further verifies that the best way to improve the quality of writing feedback is for teachers to combine their own teaching experience with generative AI. The reason behind this may be that human–machine collaboration can combine the efficiency of AI with the expertise and experience of human teachers. On the one hand, AI can generate feedback quickly; teachers, on the other hand, can adapt and complement their own expertise to provide more comprehensive and in-depth feedback. Therefore, adopting this method of man–machine collaboration will be an effective way to improve the quality of writing. Bedington et al. (2024) pointed out that man–machine collaboration has significant advantages in processing large amounts of data and performing repetitive tasks, which reduces the possibility of human intervention and error. This echoes our findings that human–machine collaboration can significantly improve the quality and efficiency of writing feedback. Professor X. Zhu (2024) gave a keynote speech entitled “Improving Pre-service Teachers’ Assessment Literacy by Using ChatGPT: A Case Study of Writing Feedback” at the Seminar of the US–China University Chinese Teachers’ Alliance, exploring how generative AI can improve the accuracy, comprehensibility, and usability of teacher feedback.

## 7. Recommendations and Limitations

### 7.1. Recommendations

In this study, we identified that it is particularly important to improve the evaluation literacy of pre-service teachers, which particularly depends on the accumulation of practical knowledge and the strengthening of professional support (Pastore & Andrade, 2019). Among the key components of evaluation literacy, feedback literacy has a direct impact on the ability of pre-service teachers to accurately grasp and effectively guide students’ learning processes in their future teaching practice (Estaji et al., 2024). In this context, generative artificial intelligence (GAI), with its unique algorithmic advantages and data analysis capabilities, provides a consistent and standardized feedback mechanism for pre-service teachers (Guénette & Lyster, 2013). Therefore, in the training process of pre-service teachers, it is essential to make full use of the technical advantages of generative AI and to build a complete feedback system that supports the comprehensive improvement of pre-service teachers’ assessment literacy. Specifically, a real-time guidance model based on GAI can be designed, where the system automatically prompts optimization strategies when teachers provide writing feedback; for example, when it detects that the feedback is too general (e.g., “Well written”), the system might suggest “Add specific details to the praise, such as ‘You used three metaphors to make the story more vivid’”. This would guide pre-service teachers to analyze the feedback generated by AI. Additionally, a low-cost pre-service teacher training system could be built using an open-source model (such as LLAMA 2) with Google Colab. By cooperating with human colleges, this system could be embedded into the existing teacher education curriculum to replace traditional composition correction practices (Xiu, 2025).

### 7.2. Limitations and Further Research

There were two main limitations in this study. First, only 11 graduate students in primary education from one university were selected. The same educational background and curriculum learning may lead to the homogeneity of participants. Therefore, follow-up research can further increase the number of pre-service teachers in different regions. Secondly, although Ernie Bot was more suitable for this study, we only took one type of generative AI as a writing feedback tool, which was a little weak; subsequent research could explore the comparison of multiple generative AIs in the future. Finally, it is important to note that this study did not verify whether pre-service teachers improve their natural (unassisted) skills in providing feedback through the process of working with AI, as cooperative feedback was carried out in the form of teacher-led AI as an assistive tool, during which pre-service teachers’ natural feedback skills could not be tested alone. In the future, a longitudinal study can be conducted to further test whether the natural feedback ability of pre-service teachers is improved in cooperative feedback.

## 8. Conclusions

Based on human–machine collaboration theory, this study compares the differences between pre-service teachers and ERNIE Bot (generative AI) in writing feedback, investigates the pre-service teachers’ willingness to use generative AI as a cooperative feedback tool, and examines the effect of human–machine collaborative feedback. It was found that Ernie Bot is significantly better than pre-service teachers in three specific feedback focuses other than language expression. Both Ernie Bot and pre-service teachers have their own advantages in writing strategies in all aspects of feedback focus and feedback strategy. All pre-service teachers showed their willingness to cooperate with generative AI in writing feedback. Finally, the human–computer collaborative writing feedback was significantly better than that of only Ernie Bot or only the pre-service teachers. Overall, these findings provide educators with a detailed blueprint with theoretical foundations and empirical validation that provide a specific and efficient operation path for the training of pre-service teachers and improve the quality of writing through more scientific and effective writing feedback strategies.

## Figures and Tables

**Table 1:** Writing focus differences between pre-service teachers and Emie Bot.

Writing Focus	Feedback	N	M	SD	F	*p*
Theme idea	Ernie Bot	45	3.311	1.856	717.548 ***	0.000
	Pre-service teacher	495	2.226	0.473		
Writing framework	Ernie Bot	45	3.333	1.871	415.927 **	0.002
	Pre-service teacher	495	2.394	0.652		
Language expression	Ernie Bot	45	2.956	1.732	77.317	0.109
	Pre-service teacher	495	2.529	0.883		
Text presentation	Ernie Bot	45	3.022	1.751	222.609 **	0.004
	Pre-service teacher	495	2.214	0.596		

Note: ** *p* < 0.01 *** *p* < 0.001.

**Table 2:** Writing strategy differences between pre-service teachers and Ernie Bot.

Writing Strategy	Feedback	N	M	SD	F	*p*
Corrective feedback	Ernie Bot	45	3.333	1.871	28.691	0.004
	Pre-service teacher	495	2.461	1.379		
Non-corrective feedback	Ernie Bot	45	2.067	1.136	18.706	0.000
	Pre-service teacher	495	3.863	0.927		

**Table 3:** Interview analysis.

Selective Coding	Axial Coding	Open Coding
Limitations of Writing Feedback by Generative AI	Lack of flexibility	Identifying errors
		Unconventional essays are not well grasped
	Lack of emotion	Feedback on emotional expression has limitations
		Evaluative language is sometimes indifferent
		Lack of encouraging words
		Some words give a sense of distance
	Lack of individuation	Recommendations are repetitive
		Some of the suggestions are not quite appropriate
		Failure to give particularly effective advice on the structure
The Advantages of Writing Feedback by Generative AI	Reducing the load	Reducing the workload of teachers
		Reducing students’ cognitive burden
	Saving time	Saving feedback time of every composition
		Reducing the overall time of feedback
		Giving students feedback quickly
	Providing specific suggestions	Giving more specific suggestions for revising
		Providing more appropriate suggestions on the theme and emotion
		Providing more optimized sentences
		Providing correct sentences for problem sentences
		Well illustrating of each student’s writing ideas
The Advantages of Cooperative Feedback	Giving emotional support	Giving warm evaluations
		Stimulating students’ writing interest due to efficient feedback
		Increasing students’ confidence in writing
	Making more comprehensive feedback	Making a more comprehensive focus
		Not only improving students’ writing but also promoting teacher development
		Generating writing ideas and recognizing scribbled writing
	Providing personalized writing feedback	Writing feedback for everyone
		Writing feedback for each essay
	Feedback is performed efficiently	Saved a lot of time for the teacher
		Achieve more with less
		Reduce the workload of teachers by 80 percent
How to Cooperate with Generative AI	The subject relationship of cooperation	Based on AI feedback, the teacher conducts a second revision
		Focus on the teacher, not only depending on AI
		Peer-to-face feedback will be given by watching the generated AI feedback with students
	Feedback process using AI	Teachers should be clear about specific points before using AI for evaluation
		In the process of using AI assessment, it complements the teacher’s own evaluation
		After the use of AI, the teacher should purposefully revise each composition again
	Timing of use	When teachers do not know how to give feedback, AI can help
		When teachers report exhaustion, AI can help
		When feedback suggestion is lacking, AI can help

**Table 4:** Pre-service teachers’ ratings for AI writing feedback.

Teacher	Writing Focus				Writing Strategy	
	Theme Idea	Writing Framework	Language Expression	Text Presentation	Corrective Feedback	Non-Corrective Feedback
F	4	5	5	3	4	4
J	5	4	4	4	5	5
L	4	4	5	3	5	4
M	4	5	5	4	5	4
Q	5	4	4	3	5	3
WYJ	5	4	5	4	4	4
WYF	5	4	4	3	5	4
x	4	4	5	4	5	2
Y	5	4	5	4	4	3
ZCF	4	5	4	3	5	3
ZXT	5	5	5	4	4	2
Mean	4.545	4.364	4.636	3.545	4.636	3.273

**Table 5:** Writing focus differences.

Writing Focus	Feedback	N	M	SD	F	*p*	Post Hoc Test (Tamhane)
Theme idea	Ernie Bot	45	3.311	1.856	256.829	0.000	Cooperation > Ernie Bot > Pre-service teacher
	Pre-service teacher	495	2.370	0.473			
	Cooperation	495	4.345	0.751			
Writing framework	Ernie Bot	45	3.333	1.871	161.131	0.000	Cooperation > Ernie Bot > Pre-service teacher
	Pre-service teacher	495	2.419	0.652			
	Cooperation	495	4.364	0.754			
Language expression	Ernie Bot	45	2.956	1.731	94.434	0.000	Cooperation > Ernie Bot > Pre-service teacher
	Pre-service teacher	495	2.352	0.883			
	Cooperation	495	4.400	0.784			
Text presentation	Ernie Bot	45	3.022	1.751	97.232	0.000	Cooperation > Ernie Bot > Pre-service teacher
	Pre-service teacher	495	2.100	0.596			
	Cooperation	495	3.564	0.631			

**Table 6:** Writing strategy differences.

Writing Strategy	Feedback	N	M	SD	F	*p*	Post Hoc Test (Tamhane)
Corrective strategy	Ernie Bot	45	3.333	1.871	45.413	0.000	Cooperation > Ernie Bot > Pre-service teacher
	Pre-service teacher	495	2.461	1.379			
	Cooperation	495	4.218	0.809			
Non-corrective strategy	Ernie Bot	45	2.067	1.136	100.757	0.000	Cooperation > Pre-service teacher > Ernie Bot
	Pre-service teacher	495	3.863	0.927			
	Cooperation	495	4.564	0.536			

**Table 7:** The time spent on feedback by pre-service teachers with and without cooperation with generative AI.

Pre-Service Teacher	Without Cooperative Feedback	With Cooperative Feedback
F	485	11
J	753	58
L	553	19
M	544	10
Q	666	39
WYJ	583	60
WYF	293	47
x	265	22
Y	437	15
ZCF	479	9
ZXT	379	10
Mean	494.273	27.273

Note: The feedback time represents the mean seconds for one composition from the pre-service teacher.

**Table 8:** Paired-sample *t*-test checking the time difference for teacher feedback with and with cooperation with generative AI.

		Paired Value			t	df	Sig. (Two-Tailed)
M	Standard Deviation	Average Value of Standard Error	Difference 95% Confidence Interval				
			Lower	Upper			
467	141.563	42.683	371.896	562.104	10.941	10	0.000

Note: The time represents the average number of minutes spent by pre-service teachers and cooperative writing feedback.

## Data Availability

The raw data supporting the conclusions of this article will be made available by the authors upon request.
